# fastp 1.0: An ultra‐fast all‐round tool for FASTQ data quality control and preprocessing

**DOI:** 10.1002/imt2.70078

**Published:** 2025-09-09

**Authors:** Shifu Chen

**Affiliations:** ^1^ LifeX Institute Gannan Medical University Ganzhou China; ^2^ HaploX Biotechnology Shenzhen China; ^3^ Faculty of Data Science City University of Macau Macau China

**Keywords:** adapter, fastp, FASTQ, filtering, preprocessing, quality control

## Abstract

Fastp has been recognized as one of the most popular FASTQ file preprocessors because of its powerful functions and extreme performance. As its first major update, fastp 1.0 will be formally presented in this paper, including its new features and the implementation principles behind it. Two other popular FASTQ preprocessors, Trimmomatic and Cutadapt, will be compared to demonstrate the great advantages of fastp in terms of simplicity, efficiency, and versatility. Some modules, such as the batch processing scripts, will be introduced on how to apply fastp to process FASTQ files efficiently. Additionally, some software design principles will be highlighted to showcase how to develop a successful bioinformatics software.

## Highlights

- Fastp is an extremely fast, full‐featured, and easy‐to‐use FASTQ data preprocessing software.
- Fastp is cloud‐friendly as it takes up very little resources and can generate web‐based reports.
- Fastp 1.0 significantly improves its HTML reporting functions and supports batch processing of many FASTQ files in parallel.
- Fastp provides users with powerful functions while maintaining efficiency and simplicity.

## Graphical abstract

The figure shows side‐by‐side quality comparison of the data before and after preprocessing using fastp 1.0. As can be seen from this figure, the data quality has been significantly improved after fastp processing. The Q20 ratio and Q30 ratio increased, and the GC base ratio returned to balance.

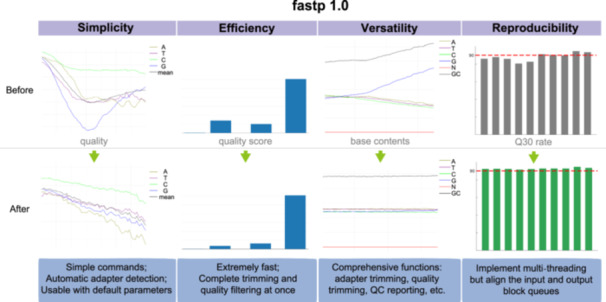

## INTRODUCTION

Sequencing technology is the core technology for exploring life sciences and conducting molecular diagnosis. Sequencing data, due to its complexity and proneness to errors, usually needs to undergo quality control and cleaning before it can enter downstream analysis. This preprocessing step usually includes performing various quality statistics on the original sequencing data, removing adapters, filtering low‐quality sequences or bases, and making some possible corrections to some sequencing errors. Many studies have shown the importance of data preprocessing in obtaining reliable analysis results. For instance, Wang et al. demonstrated that data preprocessing can significantly improve the accuracy of RNA expression analysis [1], and Bonfiglio et al. also strongly suggested FASTQ data filtering and trimming in their best practices for germline variant and DNA methylation analysis [2]. For these reasons, preprocessing of FASTQ files becomes the first and very important step in sequencing data analysis.

Due to the importance of data quality control and preprocessing, many tools have been developed to solve this problem. Among them, Trimmomatic [3], Cutadapt [4], and fastp [5] are the most widely used. Cutadapt was first published in 2011, when high‐throughput sequencing began to explode. This software has been continuously iterated and has been widely used in areas such as removing adapters and primers. However, Cutadapt is weak in terms of quality control and data filtering, and lacks some modern features such as adapter detection, splitting, merging, and UMI processing. Trimmomatic was first published in 2014. Its initial function was mainly to remove Illumina adapter sequences, and some other trimming and quality filtering functions were later added. However, Trimmomatic has not been updated in the last 5 years, since it also lacks some key features like handling UMI or performing deduplication. Furthermore, Trimmomatic and Cutadapt cannot simultaneously perform comprehensive statistical quality analysis of the data while performing adapter removal and data filtering. This often requires users to run FastQC [6] or MultiQC [7] before and after using these tools to analyze the data quality improvement. This usually results in a significant waste of computing resources and increases the cost of data analysis.

As a latecomer, fastp was first released in 2018 [8] and has been undergoing continuous development and iteration. When fastp was first designed, it was considered how to reduce costs as much as possible, including learning costs and computing costs. Therefore, fastp will simultaneously perform a comprehensive quality analysis of the data before and after preprocessing while completing filtering and trimming. At the same time, fastp's algorithm is highly optimized, so even though it provides richer functions, it's much faster than Trimmomatic and Cutadapt. In addition, fastp was designed with the use scenario in the cloud in mind, so it requires limited memory to reduce cloud server cost and provides rich HTML reports for cloud‐based web viewing.

FASTQ preprocessing has a wide range of applications in different scenarios, so software is continuously being developed. For example, BigSeqKit [9] as a parallel FASTQ preprocessing toolkit for large FASTQ data, UMIc [10] as a preprocessing method for UMI deduplication and reads correction, FastqPuri [11] as a high‐performance preprocessing software of RNA‐seq data, and FastqCleaner [12] as an interactive Bioconductor FASTQ filtering and trimming. However, this software has not yet made enough impact, possibly due to its imperfection in some aspects such as simplicity, functionality, and performance. There are also some software for miscellaneous processing of FASTQ files. For instance, SeqKit [13] and SeqKit2 [14] are great tools for FASTA/Q file manipulation, but are not specialized to preprocess data for the goal of trimming unwanted sequences and filtering unwanted reads. In this article, I mainly compare fastp with Trimmomatic and Cutadapt, as all three are the most popular FASTQ preprocessors.

## METHODS

Fastp is a multithreaded multifunctional preprocessor for FASTQ streams. Its design follows the principles of simplicity, efficiency, versatility and reproducibility. Regardless of whether the input file is single‐end or paired‐end, or what parameters are used, its multi‐threaded queue mechanism makes all results stable and reproducible.

### Simplicity

Simplicity does not mean fewer features and weaker functions, but rather providing an interface that is easy to understand while hiding its implementation details. Simplicity means anticipating the needs of the vast majority of users and making the best choice for them by default. Fastp has gone through nearly 50 minor version iterations, but has always adhered to the simplicity rule. Fastp's simplicity is reflected in many aspects. For example, its default parameters are extremely simple, requiring only input and output files to be specified, and the default parameter mode is powerful enough to meet most common scenarios. For example, in most cases, users do not need to enter the adapter sequence because fastp can detect it automatically. In fact, the algorithms for automatically detecting adapters can be very complex, but users do not need to know this.

**FIGURE 1:**
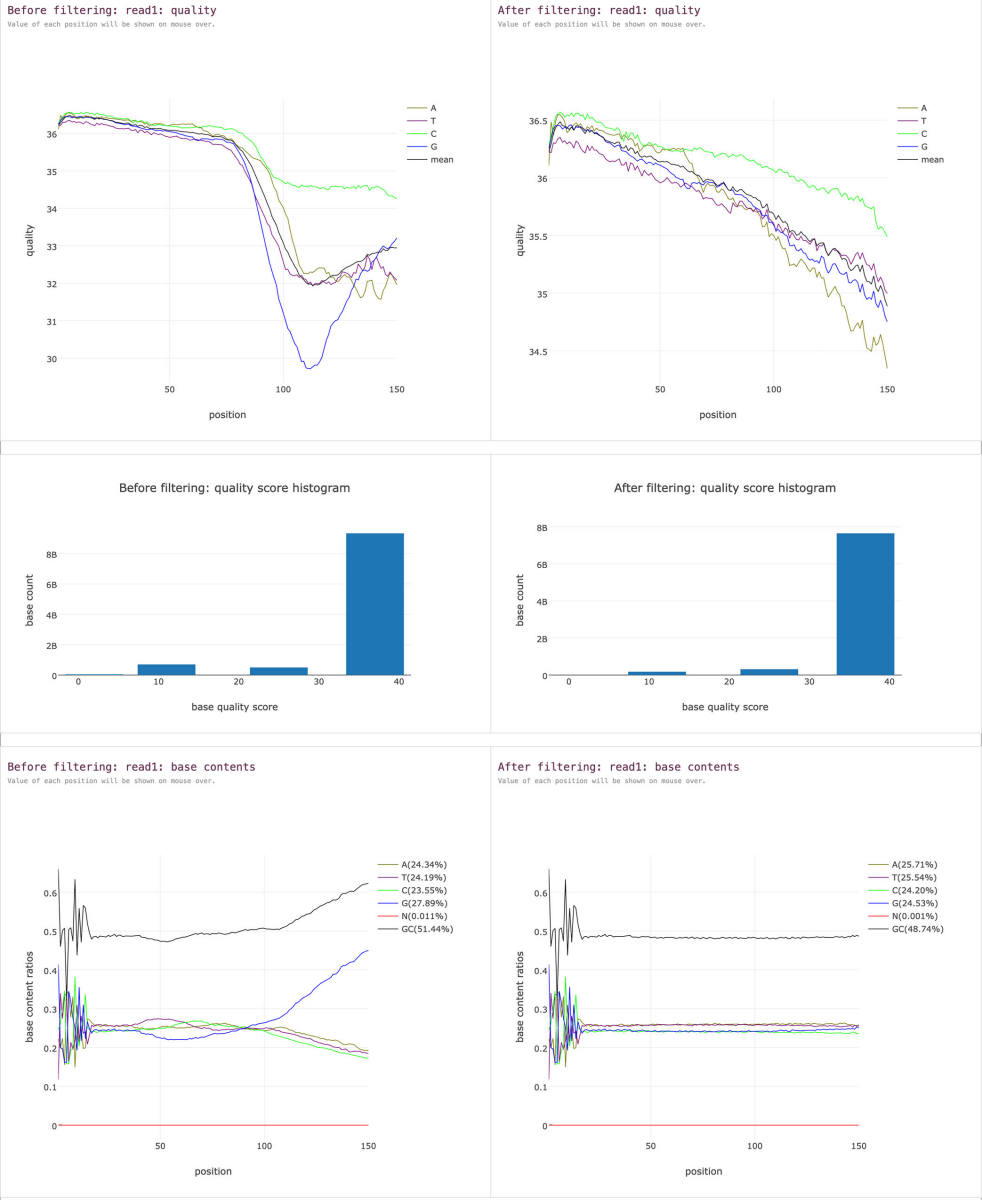
Side‐by‐side quality comparison of the data before and after preprocessing. An HTML report generated by fastp with default parameters on NCBI SRA data (SRR27128759). In this experiment, only 0.36% reads were filtered out, but many reads were cropped. As can be seen from this figure, the data quality has been significantly improved after fastp processing. The Q20 ratio and Q30 ratio increased from 93.3% to 97.8% and from 88.6% to 93.9% respectively. The GC base ratio returned to balance, from the abnormal ratio of 27.89% and 23.55% to the normal ratio of 24.53% and 24.20%. The dip in the quality curve of the original data was eliminated, and the base imbalance issue was basically solved as well.

Another example of the simplicity that fastp provides is its HTML reports. Compared to providing reports in plain text or CSV format, HTML is obviously more user‐friendly and very suitable in cloud computing environments. In fastp 1.0, the biggest change in its HTML report is that it provides a side‐by‐side comparison of pre‐processing and post‐processing data (Figure 1).

**FIGURE 2:**
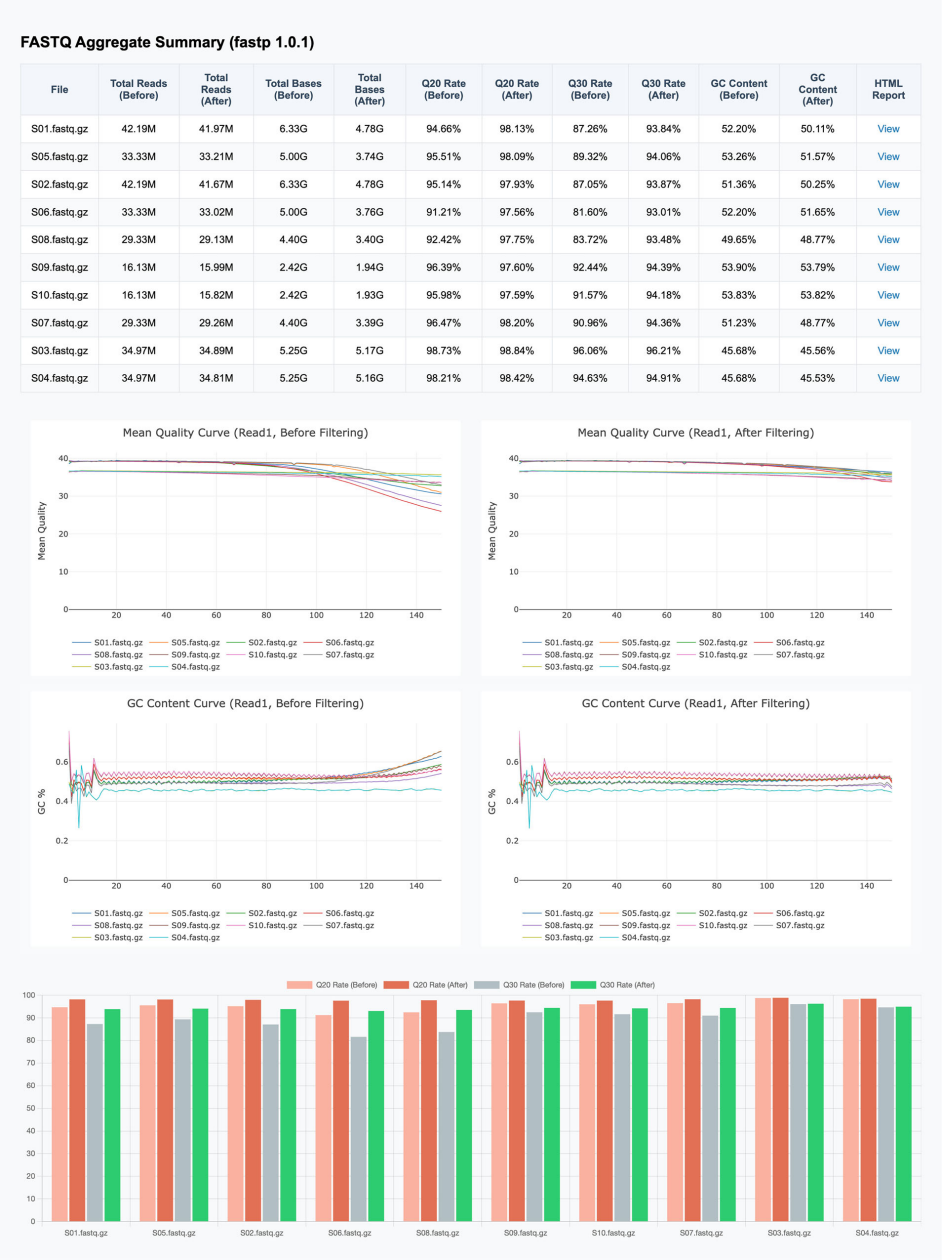
Aggregate summary of 10 single‐end FASTQ files, generated by fastp's parallel processing script. The quality of all data has been greatly improved.

In fastp 1.0, I take simplicity a step further by providing a script to batch process many FASTQ files in parallel. This script preprocesses all FASTQ files within a folder, and it can automatically couple the paired‐end FASTQ files. It generates an overall HTML report to present an aggregate summary for all processed FASTQ files. Figure 2 shows the overall HTML report of 10 FASTQ data.

### Efficiency

Efficiency usually has two meanings: high speed and low computational cost, both of which are important for bioinformatics analysis. High‐throughput sequencing files are typically very large and require a long time to complete, making computational speed crucial. Furthermore, most bioinformatics analysis processes are currently run in the cloud, requiring software to minimize CPU and memory requirements to reduce costs.

A lot of efforts have been made to improve fastp's computing speed and reduce its computing resource requirements. For example, fastp applies a simultaneous mode of filtering and performing quality analysis at the same time. It only needs to read the data once to complete the trimming and filtering of the data and obtain the quality statistics results before and after preprocessing.

Gap‐tolerant comparisons between sequences are very common to detect the presence of adapters or primers. For very short sequences, typically only one gap is allowed to guarantee the precision of comparisons. In such cases, applying sequence alignment algorithms [15] or calculating edit distances [16] would be extremely slow, resulting in significant computational overhead. In fastp 1.0, I designed a one‐gap‐matching algorithm to accelerate such operation. Simply put, for two sequences *S*_*1*_ and *S*_*2*_ of the same length *n*, while *S*_*2*_ is supposed to contain one gap to match with *S*_*1*_, I calculate the cumulative mismatch number array from left to right array as *CUML2R*, and from right to left as *CUMR2L*, respectively. Then we can quickly calculate the mismatch of *S*_*1*_ and *S*_*2*_ with one gap in *S*_*2*_ using the algorithm described below:

**Table:** 

def mismatch_with_one_gap(str_with_no_gap, str_with_one_gap):
n = len(str_with_no_gap)
CUML2R = [0] * n
CUMR2L = [0] * n
for i in range(1, n):
CUML2R[i] = CUML2R[i‐1]
if str_with_no_gap[i] != str_with_one_gap[i]:
CUML2R[i] += 1
for i in range(n‐1, 0, −1):
CUMR2L[i‐1] = CUMR2L[i]
if str_with_no_gap[i]!= str_with_one_gap[i‐1]:
CUMR2L[i‐1] += 1
min_mismatch = n
for i in range(1,n):
mismatch = CUML2R[i‐1] + CUMR2L[i]
if mismatch < min_mismatch:
min_mismatch= mismatch
return min_mismatch

The algorithm is very simple but effective in reducing the computational complexity from *O*(*n* ^*2*^) to *O*(*n*). The processing of boundaries is not shown, since it's generally handled by callers.

### Versatility

Due to the wide application of sequencing technology, the requirements for data preprocessing are also extremely diverse, which requires data preprocessors to provide a variety of functions. Table 1 shows the functional comparison among Trimmomatic, Cutadapt, and fastp. The results of this comparison referred to the documentation of this software. fastp provides most of the necessary functions (Table 1). Of course, Trimmomatic and Cutadapt are both excellent tools, and they can surpass fastp in some cases. Among them, there is a demultiplexing function that Cutadapt has, but fastp does not provide it, because I suggest that this should be the job of the demultiplexer. To maintain simplicity, some features that are less relevant were not provided.

**TABLE 1:** Comparison of features of fastp, Trimmomatic and Cutadapt.

Feature	Fastp	Trimmomatic	Cutadapt
Core purpose	Multifunctional QC and preprocessing tool with high speed	Focus on adapter/quality trimming for Illumina data	Specialized in adapter/barcode trimming with high flexibility
Adapter trimming	− Supports auto‐detection of common adapters (Illumina, Nextera, etc.) − Allows manual adapter sequence input − Trims both 5' and 3' adapters − Trim paired‐end reads by overlap analysis	− Requires explicit adapter sequences (e.g., ILLUMINACLIP parameter) − Trims 3' adapters by default; 5' trimming possible with settings − Palindrome clipping mode for Illumina paired‐end adapters	− Requires explicit adapter sequences − Supports complex adapter patterns (e.g., with mismatches, wildcards) − Trims 5', 3', or both ends
Quality trimming	− Uses sliding window quality filtering (configurable window size/quality threshold) − Trims low‐quality bases from both ends	− Implements sliding window (SLIDINGWINDOW), leading/trailing quality cuts − Well‐optimized for Illumina quality scores	− Basic quality trimming (trailing/leading low‐quality bases) − Less advanced for window‐based trimming
Read filtering	− Filters by length (min/max), N content, average quality − Removes polyG tails (common in NovaSeq)	− Filters by minimum length after trimming − Limited options for N content or average quality	− Filters by minimum length, maximum N content − Simple criteria
QC reporting	− Generates detailed HTML reports with before/after stats (length distribution, GC content, adapter content) − Visualizations (histograms, heatmaps) − Generate JSON reports	− Minimal built‐in reporting; relies on external tools (e.g., MultiQC) for QC metrics	− Basic text‐based summary and JSON reports − No visual reports
Paired‐end support	− Natively handles paired‐end data, ensuring read pairs remain synchronized after trimming	− Designed for paired‐end data; includes PE mode to maintain pair integrity	− Supports paired‐end data but requires careful parameterization to keep pairs in sync
Unique Molecular Identifier (UMI)	Supports UMI preprocessing	Not supported	Not supported
polyG/polyX trimming	Dedicated polyG/polyX tail trimming (e.g., for NovaSeq/NextSeq)	Limited poly tail trimming capabilities	polyG trimming and polyA trimming
Duplicate analysis	Supported for both single‐end data and paired‐end data	Not supported	Not supported
Insert‐size evaluation	Supported for paired‐end data	Not supported	Not supported
Deduplication	Supported for both single‐end and paired‐end data	Not supported	Not supported
Merging	Supported for paired‐end data	Not supported	Not supported
Output splitting	Supports splitting output into multiple files for parallel processing	Not supported	Not supported
Base correction	Corrects bases in overlapping paired‐end reads	Not supported	Not supported
Batch processing	Supported batch processing of multiple FASTQ files in parallel, and generate aggregate HTML report	No native support, but can be done with custom scripts	No native support, but can be done with custom scripts
Demultiplexing	Not supported	Not supported	Separate reads into different files based on sample‐specific barcodes or indexes
Usability	Simple command‐line interface with sensible defaults. Easy to configure for most common use cases	Requires familiarity with specific parameters (e.g., ILLUMINACLIP:adapter. fa:2:30:10). Slightly steeper learning curve	Intuitive syntax for basic tasks, but complex patterns require regex knowledge
Typical use cases	Comprehensive preprocessing pipelines requiring speed and detailed QC (e.g., RNA‐seq, WGS)	Illumina‐specific projects needing robust adapter/quality trimming with paired‐end data	Targeted sequencing (e.g., amplicon sequencing) with custom adapters/primers; barcode removal

### Reproducibility

Reproducibility is an important principle that many bioinformatics software developers tend to overlook. Bioinformatics software, due to its complexity and diversity, is beyond the reach of most users to understand its in‐depth details and therefore relies on the software's stable output to produce reproducible results. Fastp 1.0 provides reproducible output regardless of the input and output, the modules enabled, or the parameters used. Reproducibility is often broken due to the use of threading or random functions. Fastp implements multi‐threading, but it aligns the input and output block queues to ensure that output results are not affected by out‐of‐order execution of threads, as demonstrated.

## RESULTS

Fastp's high efficiency is one of the main reasons for its widespread adoption by the bioinformatics community. A performance evaluation experiment was conducted to compare the time taken by Trimmomatic, cutadapt, and fastp to process the same data (Table 2). To simulate common low‐cost bioinformatics cloud analysis scenarios, this experiment was conducted on a low‐profile cloud server (Tencent Cloud, 8 cores, 16 G, ubuntu 24.04), with a network cloud disk as storage. Trimmomatic was installed via Anaconda with the latest available version (0.39). Cutadapt couldn't be installed via Anaconda due to Python version conflicts, so it was installed using APT with the latest available version (4.4). All three software are configured with 4 threads, input gzipped FASTQ files, and output gzipped FASTQ files as well. In the experiment, Cutadapt removed the adapter and polyG as suggested for data of two‐color chemistry; Trimmomatic removed the adapter and performed quality trimming according to its recommended parameters. For fastp, only input/output and the threading parameters are given. However, fastp actually performed adapter trimming, quality trimming, quality filtering, length filtering, duplicate analysis, insert‐size evaluation, etc. Even though fastp performed much more analysis operations, it's still much faster than Trimmomatic and Cutadapt (Table 2).

**TABLE 2:** Speed evaluation of fastp, Trimmomatic and Cutadapt.

	S1	S2	S3	S4	S5
Fastp time (s)	165	165	138	121	66
Trimmomatic time (s)	1194	1120	1006	908	470
Cutadapt time (s)	308	200	279	241	115
Original size (G)	6.3 + 6.3	5.2 + 5.2	5.0 + 5.0	4.4 + 4.4	2.4 + 2.4
Fastp‐filtered size (G)	4.7 + 4.7	5.1 + 5.1	3.6 + 3.6	3.3 + 3.3	1.9 + 1.9
Trimmomatic‐filtered size (G)	2.1 + 2.1	4.8 + 4.8	1.0 + 1.0	1.1 + 1.1	0.94 + 0.94
Cutadapt‐filtered size (G)	4.7 + 5.7	5.1 + 5.1	3.7 + 4.2	3.3 + 3.9	1.9 + 2.2

I also employed FastQC to evaluate the data filtered by Trimmomatic, Cutadapt, and fastp, respectively. The script used in this evaluation and the FastQC results can be found at https://github.com/sfchen/fastq_preprocessor_test. From the result, we can learn that fastp‐filtered data is of very high quality, generally the best among the three. Trimmomatic‐filtered data is also of good quality, but it drops a lot of data. Cutadapt‐filtered data is of lower quality (especially for the R2 read), since it performed little besides removing adapters in this experiment.

## DISCUSSION

In this paper, I introduce the new features of fastp and the design philosophy behind it. I believe that simplicity, efficiency, versatility, and reproducibility are very important for bioinformatics, and it is recommended that bioinformatics developers follow these principles. In fact, continuing to improve simplicity, efficiency, and versatility is also the direction that fastp will continue to deepen in the future. For example, in terms of simplicity, more data types and multiple file inputs will be supported in the future. In terms of improving efficiency, fastp will introduce more SIMD technologies in the future to further improve performance and reduce computing costs. In addition, in terms of versatility, fastp will continue to iterate, while retaining ease of use, and continue to add the most requested features. The changelog of fastp from v0.1 to v1.0 can be found in Table S1. For end‐users and bioinformatics community contributors, it's suggested to keep watch on fastp's repository (https://github.com/OpenGene/fastp) to follow up on minor version updates.

## AUTHOR CONTRIBUTIONS

**Shifu Chen**: Conceptualization; investigation; funding acquisition; methodology; validation; visualization; writing—original draft; writing—review and editing; software; formal analysis; project administration; data curation; supervision; resources.

## CONFLICT OF INTEREST STATEMENT

The author declares no conflicts of interest.

## ETHICS STATEMENT

No animals or humans were involved in this study.

## Supporting information


**Table S1:** Changelog of fastp from v0.1 to v1.0.

## Data Availability

The data that support the findings of this study are openly available in fastp at https://github.com/OpenGene/fastp. The fastp software is a part of the OpenGene project. Its source code and prebuilt binaries can be found at https://github.com/OpenGene/fastp. Supplementary materials (tables, graphical abstract, slides, videos, Chinese translated version, and update materials) may be found in the online DOI or iMeta Science http://www.imeta.science/.
