# Supplementary material for: fastp 1.0: An ultra‐fast all‐round tool for FASTQ data quality control and preprocessing
Source: Imeta. 2025 Sep 9;4(5):e70078. doi: 10.1002/imt2.70078 (PMC12527978; doi:10.1002/imt2.70078)
Supplement: Supplementary file 1 — Table S1: Changelog of fastp from v0.1 to v1.0. [file IMT2-4-e70078-s001.docx]

**fastp changelog**

| **version** | **date** | **change** |
| --- | --- | --- |
| **v1.0** | 2025-06-15 | support batch processing in parallel.  add quality histogram figure  add Q40 to statistics  allow up to 64 threads |
| **v0.26** | 2025-06-05 | support searching adapters with gaps  refine single-end adapter detection by searching known adapter first |
| **v0.25** | 2025-06-01 | support side by side HTML report  handle weird FASTQ files  make interleaved output reproducible |
| **v0.24** | 2024-11-07 | improve memory usage stability  add QIASeq miRNA adapter sequence to known ones  update plotly.js URL to HTTPS  use SingleProducerSingleConsumerList to remove mutex and get reproducible result |
| **v0.23** | 2021-10-08 | generates reproducible outputs and improves performance greatly  switch to isa-l for gzip decoding |
| **v0.22** | 2021-08-24 | support deduplication  add alternative plotly https src |
| **v0.21** | 2020-06-19 | support outputing overlapped regions to get the cleanest data  support MGI FASTQ ID format |
| **v0.20** | 2019-04-17 | revise overlap detection, PE correction and adapter trimming  support average quality score filter  count polyX |
| **v0.19** | 2019-04-09 | support trimming multiple adapters  outputting unpaired/failed reads  support -m/--merge option to merge paired reads add detect_adapter_for_pe option to allow adapter detection for PE data  support trim to max_len  improve adapter trimming for the reads like adapter dimers |
| **v0.18** | 2018-07-05 | improve speed and compression rate  improve duplication estimation improve output splitting report mean length |
| **v0.17** | 2018-06-27 | support insert size estimation  improve adapter detection |
| **v0.16** | 2018-06-20 | supports STDOUT streaming and interleaved input |
| **v0.15** | 2018-06-02 | support duplication evaluation  add --dont_overwrite option |
| **v0.14** | 2018-05-14 | supports quality preview and index filtering  support limit the number of reads to process  support index filtering |
| **v0.13** | 2018-04-17 | improve adapter detection  improve low complexity filter  perform polyG --> polyX trimming in 3'ends when they are both enabled |
| **v0.12** | 2017-12-13 | support overrepresentation analysis  evaluate sequencing length |
| **v0.11** | 2017-12-07 | support KMER counting  show GC content in summary table  show base content percentage in base content graph |
| **v0.10** | 2017-12-05 | support polyG tail trimming  make prefix configurable for UMI, and no prefix for default |
| **v0.9** | 2017-11-29 | support UMI processing  add option to change the report title |
| **v0.8** | 2017-11-26 | support splitting by lines |
| **v0.7** | 2017-11-23 | support base correction by overlap analysis |
| **v0.6** | 2017-11-20 | support long reads (PacBio / Nanopore)  optimize plotting for long reads using down sampling |
| **v0.5** | 2017-11-10 | detect adapter sequence automatically for SE data |
| **v0.4** | 2017-11-09 | add adapter trimming for single end data  make length filtering default |
| **v0.3** | 2017-11-08 | add sliding cut by quality in 5' and 3'  support phred64  show N in content curves |
| **v0.2** | 2017-11-08 | support splitting to multiple output files  change argument for threading  use different arguments for read1/read2 trimming |
| **v0.1** | 2017-11-06 | the first release! |
